# Limited Use of Price and Quality Advertising Among American Hospitals

**DOI:** 10.2196/jmir.2660

**Published:** 2013-08-29

**Authors:** David B Muhlestein, Chrisanne E A Wilks, Jason P Richter

**Affiliations:** ^1^Division of Health Services Management and PolicyThe Ohio State University College of Public HealthColumbus, OHUnited States

**Keywords:** hospitals, patients, quality indicators, commerce

## Abstract

**Background:**

Consumer-directed policies, including health savings accounts, have been proposed and implemented to involve individuals more directly with the cost of their health care. The hope is this will ultimately encourage providers to compete for patients based on price or quality, resulting in lower health care costs and better health outcomes.

**Objective:**

To evaluate American hospital websites to learn whether hospitals advertise directly to consumers using price or quality data.

**Methods:**

Structured review of websites of 10% of American hospitals (N=474) to evaluate whether price or quality information is available to consumers and identify what hospitals advertise about to attract consumers.

**Results:**

On their websites, 1.3% (6/474) of hospitals advertised about price and 19.0% (90/474) had some price information available; 5.7% (27/474) of hospitals advertised about quality outcomes information and 40.9% (194/474) had some quality outcome data available. Price and quality information that was available was limited and of minimal use to compare hospitals. Hospitals were more likely to advertise about service lines (56.5%, 268/474), access (49.6%, 235/474), awards (34.0%, 161/474), and amenities (30.8%, 146/474).

**Conclusions:**

Insufficient information currently exists for consumers to choose hospitals on the basis of price or quality, making current consumer-directed policies unlikely to realize improved quality or lower costs. Consumers may be more interested in information not related to cost or clinical factors when choosing a hospital, so consumer-directed strategies may be better served before choosing a provider, such as when choosing a health plan.

## Introduction

Numerous policies intending to improve the health care system in the United States have been proposed and some have been adopted. Some policies, called consumer-directed strategies, have attempted to change the health care system by targeting the behavior of the consumer of services. For instance, in 2003, health savings accounts and high-deductible insurance plans were embraced with the intent to encourage consumers to be price conscious at the time of service [1,2]. Some states have mandated some transparency in hospital pricing to encourage this behavior [3,4]. These strategies suppose that price-conscious consumer behavior will lead to providers increasing health care value in the form of better quality or lower prices [5]. For the policymaker’s consumer-based system to increase value, there are 3 criteria that must be met: (1) consumers must have access to information on costs and quality, (2) they must choose providers based on those factors, and (3) providers must compete with one another to lower costs or improve quality [6].

There is no guarantee, however, that a consumer-directed system will necessarily lead to hospital competition on price or quality. Even if valid measures of price and quality are available, consumers must still choose hospitals based on these factors. Choice of providers, unlike purchasing fungible goods in the marketplace, is dictated by many nonprice factors, including insurance status, physician recommendations, location, institutional perception, and patient experience. When choosing insurance, cost is an important factor, but once the insured party becomes ill, other factors not related to price or quality are likely to dominate, such as proximity to family or prior relationship with a physician. If consumers are able to direct their care but choose providers based on considerations not related to price or quality, a consumer-directed policy will not lead to increased value.

For a consumer-directed system to lead to improved value, hospitals, which represent the largest proportion of American health care spending, must be responsive to needs and choices of consumers [7]. If hospitals currently are responsive to consumer preference for lower prices and higher quality, there will be evidence that they are competing for consumers based on price or quality. If hospitals are not competing on price and quality, but information on these factors is made available, there is the possibility that hospitals could compete on these factors to increase consumer value in the future. This study will identify whether hospitals are currently competing on price and quality and, if not, what factors they are competing on.

## Methods

### Overview

It is estimated that 78% of Americans currently use the Internet, that 80% of Internet users compare health care options online, and 58% use it to obtain health information [8-10]. Websites are a nearly universally accessible resource that consumers have to compare services. Because of the ease of access and the prevalence of consumers using the Internet to obtain health care information, hospital websites represent a good source of information to determine what information on price and quality is available to consumers and identify which factors hospitals use to compete for individual consumers. This study presents findings from a review of American hospitals’ use of advertising on their company websites. No dataset exists which has systematically evaluated the approaches that hospitals use on their websites to advertise to individual patients; therefore, we performed an original evaluation on how hospitals use varying approaches to entice consumers to use a facility.

### Data Collection

The sampling frame for this study was all Medicare-registered hospitals as of July 2012 [11]. This represents 4739 hospitals from all 50 states, the District of Columbia, and several United States territories. Hospitals that register with Medicare include acute care, critical access, and children’s hospitals as well as hospitals administered by the Department of Veterans Affairs (VA). We used a 10% simple random sample of these hospitals, resulting in a sample size of 474 hospitals. Information on the hospitals included in our sample and on our sampling frame can be found in Table 1. Information on bed size was obtained by matching hospitals to Centers for Medicare and Medicaid Services (CMS) Healthcare Cost Report Information System (HCRIS) data. For sampled hospitals, we researched hospital websites for missing values (VA hospitals do not submit CMS cost reports). For the total sample, missing bed sizes were excluded. A hospital was considered urban if it was located in a metropolitan area of a core-based statistical area (CBSA), indicating a regional population of at least 50,000 (determined using a zip code-to-CBSA crosswalk).

The 474 hospitals were divided among the 3 authors and were reviewed between July and September 2012. To insure uniform review of the websites, the reviewers initially evaluated several websites as a group to come to a consensus on evaluating site elements. To test for agreement, multiple websites were reviewed independently by the reviewers; Fleiss’ kappa values indicate substantial agreement among all reviewers on identifying categories (kappa=0.633) with slightly lower agreement (kappa=0.571) among specific levels within categories [12]. Any hospitals that did not have a website were confirmed by at least 2 reviewers. Some hospitals that are part of systems do not have dedicated websites, but instead have a webpage as part of the system’s website. We used the hospital’s webpage for our analysis unless it was so basic as to not mention any services the hospital provides; in these cases we used the system’s website (6.1%, 29/474 of hospitals).

### Website Evaluation

The focus of our research was on the home page of the website where website visitors are most likely to reach first when researching a hospital. On each reviewed website, the authors sought to identify each of the means in which hospitals may attract customers and refer to these means collectively as forms of advertising. These include formal advertising, such as banners for specific service lines, as well as other content, such as patient education material, that may entice a consumer to use that hospital. Specifically, we sought to identify content related to cost, quality, price, patient safety, customer satisfaction, personal stories, amenities, service lines, access, technology, research, awards, patient education, affiliations, and employment opportunities. Table 2 contains descriptions of these categories of content. Content could fall into more than 1 category, such as an advertisement for 3D mammography representing both technology (the 3D equipment) and a service line (radiology).

After a category of content was identified, it was then classified based on the prominence of the information on the home page as the page’s major focus, a minor focus, or a link. The major focus is the primary content in the body of the page, generally including a header and graphical elements or pictures; it is often near the top-center of the page and it is what the eye is usually first drawn to. There can only be 1 major focus on a page, but the major focus can rotate through a number of individual topics, in which case we captured all the categories of content shown on the rotating image. A minor focus is on the home page and includes pictures or content smaller in size compared to the major focus but which contains more than just a link; there can be multiple minor foci on a page. The final category includes text links that navigate the reader to another page. Links may be constantly visible on the website or accessible only through drop-down menus. Reviewers were also able to add, in a free response section, any comments about the individual categories or the overall website.

In addition to content on the home page, we hypothesized that consumers who were interested in price and quality information would search on a page to find more information. On sites that had a search feature, we additionally searched for the following terms: cost, price, quality, and patient safety. We then clicked on any link on the first page of results that had the search term in the title or preview and checked the page that was linked to for content relating to price, quality, or patient safety.

**Table 1:** Summary statistics of the hospitals included in this study.

Characteristic		Sample (N=474)		All US hospitals, % (N=4739)
		n	%	
Hospital is still in operation		472	99.6	
Hospital has a website		453	95.6	
**Hospital region**				
	New England	19	4.0	4.0
	Middle Atlantic	41	8.6	9.0
	East North Central	82	17.3	15.6
	West North Central	57	12.0	13.6
	South Atlantic	67	14.1	14.6
	East South Central	43	9.1	8.5
	West South Central	70	14.8	14.6
	Mountain	49	10.3	7.9
	Pacific	42	8.9	11.0
	Associated Areas	4	0.8	1.2
**Urban/rural status**				
	Urban	277	58.4	60.4
	Rural	197	41.6	39.6
**Number of beds**				
	<25	77	16.2	17.3
	25-49	104	21.9	22.5
	50-99	80	16.9	16.3
	100-199	105	22.2	22.6
	200-299	56	11.8	10.8
	300-399	23	4.9	5.6
	400-499	15	3.2	2.3
	500+	14	3.0	2.5
**Hospital ownership**				
	Nonprofit	268	56.5	58.0
	For profit	92	19.4	16.9
	Government	114	24.1	25.0
**Hospital type**				
	Acute care	351	74.1	73.3
	Acute care veterans administration	13	2.7	2.7
	Children’s	1	0.2	0.5
	Critical access	109	23.0	23.5

**Table 2:** Categories of advertising on hospital websites.

Category	Description
Price	Any information on the price or cost of services. This includes information on the price for specific procedures or services as well as information on the average cost of services for a particular diagnosis. The information could deal with the list prices, average reimbursed price, or the expected copayments of the patient. This could be located on the home page or found via a website search.
Quality outcomes	Quantifiable information on outcomes of care. These include data on process or outcome measures, or comparison statistics in which the hospital’s outcomes are presented relative to other hospitals. Qualitative descriptions of quality were not included in this category, such as a statement that the hospital is “a regional leader in cardiovascular outcomes,” unless accompanied by some quantifiable data. This could be located on the home page, found via search, or linked to offsite. An example of this occurs when a hospital publishes its own scores on 30-day readmissions from Medicare’s Hospital Compare website or informs the consumer about data available from an offsite source and links the consumer to the external website.
Patient safety	Quantifiable depiction of patient safety outcomes. This includes information on rates of hospital-acquired infections, pressure ulcers, medication errors, falls, surgical errors, etc. Information could be found on the home page or via search; data could be located on the website or available via a link to an external website.
Customer satisfaction	Data on previous patients’ experiences with the hospital. This could include information from Hospital Consumer Assessment of Healthcare Providers and Systems (HCAHPS) surveys, Press Ganey patient satisfaction surveys, or some other numerical or comparative depiction of patient satisfaction with care. An example is the percent of patients that would recommend the hospital to their friends or family.
Awards and accreditation	Formal recognition from an outside entity. Examples include being a top hospital in some specialty or receiving accreditation for a service.
Personal stories	Anecdotal experiences from patients or staff that recount the care they received or provided at the hospital. This includes written experiences and videos relating to how the hospital served the patient or the experience of staff while working at the hospital and serving patients.
Amenities	Includes references to the physical facilities, such as buildings, parking, cafeterias, and gift shops or support services, such as consumer advocates, chaplains, and support groups. Also includes qualitative descriptions of the hospital, such as the atmosphere patients will experience, and nonmedical information available at the hospital, such as cooking classes or healthy recipes.
Service lines	Specialties and service lines that the hospital offers; includes inpatient and ambulatory services.
Access	Features of the hospital that make receiving care easier for patients. This can be in the form of some convenience (location or hours of operation), insurances accepted, lists of physicians affiliated with the hospital, ways to interact with the hospital through social media, and online features, such as emergency department wait times and online bill pay.
Technology	Medical technology or equipment the hospital uses. Examples include robotic surgery and 3D mammography.
Research	Any form of inquiry into outcomes of disease. Includes formal clinical trials under review at the hospital and any highlighted research of individual staff members.
Patient education	Any information for patients on diseases, such as prevention, treatment, or disease management; includes classes, videos, and risk assessment guides in addition to literature.
Affiliations	Formal relationships with universities or hospital systems. This only includes hospitals that advertise an affiliation with a university or hospital system on their website.
Employment opportunities	Information on available jobs at the hospital, including job listings and online job applications.

## Results

We estimated the percent of hospitals in the United States that use various categories of advertising on their website. We present whether the hospital had a focus on the category (indicated by having either a major focus or a minor focus) and whether there was any content relating to the category (major focus, minor focus, link, and search results, if applicable). Our findings are presented in Table 3.

The major finding was that very few hospitals focused on price or quality information. Only 1.3% (6/474) of hospitals had a price focus on their website, 5.7% (27/574) had a quality focus, and 3.2% (15/474) had a patient safety focus. Instead, hospital website advertising was geared more toward service lines (56.5%, 268/474), access (49.6%, 235/474), awards (34.0%, 161/474), and amenities (30.8%, 146/474). The most common form of advertising that hospitals had was information on employment opportunities (92.6%, 439/474), which is not directly applicable to patients.

Having some information on price, however, did not mean that patients had full access to price. Of the 6 hospitals that had any price information in their focus area, only 1 had it as a major focus, although that major focus was limited to the price of 1 procedure (a $99 calcium score screening). Of the 5 hospitals with a minor focus on price, 3 had information on cost estimates or ranges of common prices for common procedures, 1 had the price of 2 weight loss procedures, and 1 had the price of 1 heart scan.

Quality outcomes information was accessible on 40.9% (194/474) of websites. The information, however, was generally minimal and was often difficult to find or interpret on the website. Only 3 hospitals used quality data as their major focus, with 2 referencing Medicare’s Hospital Compare data and the third, a cancer specialty hospital, showed cancer survival rates. Hospitals that included quality data often cited Hospital Compare data, but provided no comparison to other local hospitals. Others only provided data on a subset of outcomes measures, such as a general hospital only providing cardiovascular outcomes measures. Further, only 23.0% (109/474) of all hospitals had quality outcomes measurements on their site, with the remainder linking to external sources, primarily Hospital Compare or state quality reporting sites [13]. Often, data on quality were available if a consumer was willing to look for it, but hospitals were not actively competing on it, with 5.7% (27/474) having it as a major or minor focus.

Patient safety information was much less common than quality information, with only 20.3% (96/474) of hospitals having any such information available. The most common data was Hospital Compare data or Hospital Safety Score information from the Leapfrog Group [14]. In all, 44.8% (43/96) of hospitals with patient safety information linked to offsite data. Patient safety was mentioned qualitatively at least as often as quality, but quantitative data were much less common; we suspect this is because no hospital wants to show data indicating they are unsafe for any of their patients.

The most common patient-directed advertisement method was to emphasize specific service lines the hospital offers; 56.5% (268/474) of hospitals focused on 1 or more service lines and 93.5% (443/474) had some information on service lines on their home page. A list of the specific service lines that hospitals had a major focus on is available in Table 4. The most common service lines to receive a major focus included specialties related to heart disease, cancer, women’s services, and orthopedic surgery, which are often considered to be profit centers for hospitals [15]. Access (49.6%, 235/474) and amenities (30.8%, 146/474) were also very common focus categories, whereas customer satisfaction was not (2.7%, 13/474). These categories were focused around how the experience of care would be for the patients, as opposed to the actual reported experience of care.

**Table 3:** Percent of US hospitals that use various types of website advertising (N=474).

Type of advertising		Focus		Any information	
		n (%)	SE %	n (%)	SE %
**Price**		6 (1.3)	0.5	90 (19.0)	1.7
**Quality**					
	Quality outcomes	27 (5.7)	1.0	194 (40.9)	2.1
	Patient safety	15 (3.2)	0.8	96 (20.3)	1.8
	Customer satisfaction	13 (2.7)	0.7	83 (17.5)	1.7
	Awards and accreditation	161 (34.0)	2.1	222 (46.8)	2.2
**Other factors**					
	Stories	83 (17.5)	1.7	117 (24.7)	1.9
	Amenities	146 (30.8)	2.0	309 (65.2)	2.1
	Services	268 (56.5)	2.2	443 (93.5)	1.1
	Access	235 (49.6)	2.2	419 (88.4)	1.4
	Technology	112 (23.6)	1.9	140 (29.5)	2.0
	Research	16 (3.4)	0.8	62 (13.1)	1.5
	Patient education	157 (33.1)	2.1	356 (75.1)	1.9
	Any university affiliation			25 (5.3)	1.0
	Any health system affiliation			233 (49.2)	2.2
	Any employment information			439 (92.6)	1.1

**Table 4:** Hospital websites with a major focus on service lines.

Major service line		n	% of total hospitals (N=474)	% of hospitals with any major focus service line (n=170)
**Hospitals with any major focus service line**		170	35.9	100
**Specific service lines**				
	Bariatric/weight loss/eating disorders	21	4.4	12.4
	Cardiovascular disease	72	15.2	42.4
	Emergency medicine	21	4.4	12.4
	Labor and delivery	9	1.9	5.3
	Multiple services	11	2.3	6.5
	Neurology/neurosurgery	15	3.2	8.8
	Obstetrics/gynecology	39	8.2	22.9
	Oncology/cancer care	44	9.3	25.9
	Orthopedic surgery	33	7.0	19.4
	Other service line	54	31.8	11.4
	Pediatrics	12	2.5	7.1
	Radiology	13	2.7	7.6
	Surgery	21	4.4	12.4
	Wound care	9	1.9	5.3

## Discussion

### Overview

With so few hospitals focusing on price and quality information, it is apparent that hospitals are not actively competing for individual patients based on these factors. For a value-driven, consumer-based health system to hope to function, information on price and quality must be available. In our present system, however, there is insufficient information available for a consumer that wants to be engaged to adequately compare their options, rendering a consumer-driven system in its current form unviable.

### Price Information

Nearly 19.0% (90/474) of hospitals do have some price information available on their website, but the information that is available is limited. Ohio, which had the most hospitals with some price information (86.4%, 19/22), has a law that requires hospitals to post price information on their websites, but this is limited to daily room charges and hospital charges for the 30 most common services in a variety of departments [3]. There are multiple challenges, however, with this law. First, the statute does not require hospitals to include information on physician services, supplies, or other nonhospital charges the patient may be billed for during their hospitalization. Second, it is limited to the specific hospital’s most common procedures; therefore, the procedure a patient may be interested in may not be included on a hospital’s charge list. Third, the service descriptions use technical language and there is no requirement to define the terms; for example, a patient comparing emergency department charges would have no direction how to estimate what level (1 through 5) their issue may be classified as or whether their need may require more or less than 31 to 74 minutes of critical care. Fourth, and very significantly, there is no requirement to differentiate between charges and reimbursement rates. Hospitals charge rates have grown extensively over the past decades and significantly differ from actual payments that payers make for services rendered [16,17]. With no breakdown of what a patient may expect to pay, particularly in relation to their insurance, there is no way for consumers to appropriately compare costs.

Consumers are unable to adequately compare prices when fewer than 1 in 5 hospitals has any information on prices available. Of the hospital websites that do contain information on price, the information is limited to charges. No hospital provided information by insurer on how much a patient’s out-of-pocket or total costs may actually be. Calculating out-of-pocket estimates may be difficult, but all hospitals have ready access to their chargemaster (a record of prices for all billable services) and, for most insurers, hospitals have ready access to the negotiated reimbursement rate for each service. At a minimum, hospitals could provide information on how much Medicare copays may be because that represents a large portion of their patient population with known copays. The information is available; it is just not being shared.

If this information is readily available to hospitals, why is it not available to patients? There are many potential reasons that a hospital may not share price information, including already having a ready supply of patients and because patients are not actively seeking this information. Although some hospitals are in competitive markets, many are in areas where they are the only hospital within comfortable travel distance, effectively giving them a regional monopoly on hospital services. Because rural hospitals are in less-populated areas, it is expected that they would also be in less-competitive markets as fewer hospitals will be close together. If this is the case, we would expect urban hospitals to be more likely to have price information than rural hospitals. Although we found this to be the case (urban 22.0% vs rural 14.7%, *P*=.03), even urban hospitals appear to have little need to share price information. There may not be pressure on hospitals to provide this information because most patients may not care due to their health insurance shielding them from the effects of the price variation [18]. Patients may also rely on physicians to make hospital choices for them.

There are also reasons that may actually discourage hospitals from sharing information, including not wanting to hurt their bargaining position with insurers and because it limits their ability to price discriminate among individual patients. It is known that there are wide discrepancies between and within markets as to what hospitals are reimbursed for similar services [19-21]. If actual prices paid by different insurers were available, hospitals’ negotiating power with those insurers would likely be weakened and their ability to price discriminate while negotiating with insurers or with individual purchasers would be diminished [22,23].

### Quality

Similar to price, hospitals are not directly competing on quality outcomes. Although information on general outcomes is readily available through Hospital Compare, hospitals are not actively advertising their quality outcomes data to their patients. Hospitals attempt to convey quality via proxy measures or may feel no need to compete for patients based on quality.

A proxy representation of quality is anything that will imply a high level of care and good outcomes. This can be done in multiple ways, including advertising specific service lines, referencing external reviews of the facility, and by advertising technological advances. The most common approach is to focus on a specialty and add qualitative descriptions of how high quality outcomes and patient satisfaction are achieved.

A second proxy for quality is awards. These represent external recognition of the hospital, usually involving a specific service line, and assumes the external reviewer, because of an ability to evaluate the hospital’s performance in a way that average consumers cannot, is in a position to make an objective pronouncement on the hospital’s quality. Awards, however, have been criticized for not correlating well with objective outcomes and for methodological problems, such as being biased toward reputation [24,25]. Hospitals often focus on awards (34.0%, 161/474) and nearly half (46.8%, 222/474) mention them. Table 5 contains a breakdown of common awards. Other awards commonly cited included specialty society accreditations, local business awards (such as “Best Places to Work”), “Most Beautiful Hospital” awards, and others. Hospitals that listed any awards mentioned 4.6 different awards, on average.

Another proxy is use of technology. Although some technological advancements do improve care, others have not been shown to lead to better clinical outcomes while costing more [26,27]. Whether technological innovations always justify the costs is debated, but technology’s ability to attract patients is well known [28,29].

Another possibility for the dearth of quality outcome focus is that hospitals do not feel that the information is a priority for most patients. If they felt that some patients were interested and they wanted to compete for these patients, then the information would be made available, but it would not be a focus. If this were the case, hospitals that are in areas that are more competitive would be more likely to have some quality information than those in less-competitive markets. Indeed, urban hospitals are much more likely to provide any quality information than rural hospitals (urban: 50.2%, 138/277; rural: 27.9%, 55/197, *P*<.001).

**Table 5:** Awards listed on hospital websites.

Award	% of total hospitals (N=474)	% of hospitals with any awards (n=222)
Any award	46.8	100.0
US News	8.6	18.5
Magnet	8.0	17.1
Leapfrog Group	3.2	6.8
Joint Commission	16.0	34.2
Thomson Reuters	6.1	13.1
Other awards	33.5	71.6

### What Are Hospitals Competing On?

We grouped hospitals into 2 categories: those that do have an advertising focus (excluding affiliation or employment opportunities) and those that do not. In all, 84.0% (398/474) of hospitals do have some advertising focus on their website. Hospitals without a focus may not be competing for individuals at all. With narrowing networks, patients will tend to go to hospitals where their insurance is accepted, meaning the responsibility to evaluate costs and quality is relegated to the insurer [22,30]. Although insurers are undoubtedly interested in quality outcomes at the population level when negotiating with providers, their primary interest is minimizing population costs, given their purchasing power [31]. This, however, negates the potential impact of individual consumer-directed care as the responsibility is moved to third-party insurers.

Of the hospitals that do have some advertising focus, 89.6% (354/398) focus on service lines, access, or amenities. A focus on services (67.1%, 267/398 of hospitals with any focus) implies that the service line, among the others at the hospital, is exceptional. Without associated data on why it is exceptional, such as quality or cost information, the hospital is not competing for the value of the service, but the brand of the service. A focus on access or amenities (72.1%, 287/398) speaks to the experience of care, such as ease of receiving services or quality of facilities. The experience of care is 1 of the triple aims mentioned by the Institute for Healthcare Improvement, but this experience of care is not generally associated with better outcomes or lower costs [32,33].

This focus on patient experience, however, does make sense because that represents what many consumers are primarily interested in. A survey of commercially insured patients found the most important factor in choosing a hospital is patient experience, which was more than twice as important as clinical reputation [34]. Another study found that an increase in amenities, such as good food and attentive staff, lead to a significantly greater demand for the hospital among patients [35,36]. In recent years, there has also been unprecedented growth in patient experience-focused hospitals [37]. It is unclear whether increasing access to price and quality information will lead to significant changes in consumer preferences at the point of service as other factors are likely more important, such as established physician relationships, location, and the amenities of the hospital where the patient will stay. Immediately before a hospitalization, particularly when a patient is either in an emergency or suffering from the effects of a chronic condition, is not an ideal time to require patients to actively compare quality and price values between hospitals.

A health care system that increases value may not be achievable via a consumer-directed design at the point of service if consumers are more interested in factors not related to price or clinical outcomes immediately before choosing a hospital. A better approach to increase value is to redirect the consumer-based designs away from the point of service of care and instead incent consumers to purchase insurance based on lower prices and higher quality. This can be accomplished by increasing price and quality information relating to the networks that insurers have negotiated with. If patients can be steered toward lower-cost, higher-quality providers before they are ill and are generally satisfied with their care, then they are likely to continue with that provider [38]. Focusing consumer-based reforms on the point of service may be too late to achieve meaningful improvements in clinical outcomes and decreases in health care costs.

### Limitations

A limitation of this study is that it is focused on information available on hospital websites. There remains the possibility that hospitals have price or quality data accessible to patients at another source that is convenient to use, but is not mentioned on their websites. We feel that this is unlikely, but it is a possibility. Given the high proportion of consumers that use the Internet for health comparisons, we feel this limitation is minor. It is more likely, however, that hospitals advertise apart from their website (print, television, billboards, etc). Our findings are thus limited to advertisements on the hospitals’ own websites.

### Conclusions

For a consumer-driven health care system to lower health care costs and better health outcomes, information on price and quality must be available, consumers must choose providers based on those factors, and then providers must compete to improve on price and quality. There currently is not adequate information available for consumers to compare prices. Further, the minimal price information that exists is insufficient for a consumer to estimate how much their care may actually cost them out-of-pocket. There is more information available to consumers on quality measures from third parties, but hospitals are not actively competing on clinical outcomes of care. Hospitals do, however, compete on proxies for quality, including awards and by advertising medical technology, but these proxies do not always correlate with improved clinical outcomes.

Rather than prices or quality, hospitals are primarily competing on patient experience factors, such as amenities and conveniences. This may be because consumers are more interested in the experience of care at the time they are sick. A better approach is to encourage patients to choose low-cost, high-quality providers much earlier, such as when they purchase health insurance, rather than waiting until they are sick.

## Abbreviations

CBSA: core-based statistical area
CMS: Centers for Medicare and Medicaid Services
HCAHPS: Hospital Consumer Assessment of Healthcare Providers and Systems
HCRIS: Healthcare Cost Report Information System
SE: standard error
VA: Veterans Affairs
